# EZH2 Regulates the Proliferation-Senescence Balance and Tumor–Stromal Signaling in Lung Adenocarcinoma

**DOI:** 10.3390/ijms27135914

**Published:** 2026-06-30

**Authors:** Kamil Saramowicz, Matylda Piesiewicz, Angelika A. Adamus-Grabicka, Pengyu Zhao, Joanna Sikora, Wioletta Rozpędek-Kamińska

**Affiliations:** 1 Department of Clinical Chemistry and Biochemistry, Medical University of Lodz, 92-215 Lodz, Poland; kamil.saramowicz@stud.umed.lodz.pl (K.S.); matylda.piesiewicz@stud.umed.lodz.pl (M.P.); 2 Department of Bioinorganic Chemistry, Medical University of Lodz, 90-151 Lodz, Poland; angelika.adamus@umed.lodz.pl; 3 College of Life Science and Technology, Huazhong University of Science and Technology, Wuhan 430074, China; pengyu122601@hust.edu.cn; 4 Key Laboratory of Molecular Biophysics of the Ministry of Education, Wuhan 430074, China

**Keywords:** EZH2, lung adenocarcinoma, cellular senescence, senescence-associated cytokines, tumor microenvironment, cancer-associated fibroblasts, epigenetic therapy, tazemetostat, MS1943, targeted protein degradation

## Abstract

Enhancer of zeste homolog 2 (EZH2), the catalytic component of the polycomb repressive complex 2, is frequently overexpressed in lung adenocarcinoma and contributes to transcriptional programs that support tumor proliferation and cellular plasticity. However, its role in regulating senescence-associated signaling and tumor–stromal interactions in lung cancer remains incompletely understood. In this study, we combined transcriptomic analysis of The Cancer Genome Atlas lung adenocarcinoma cohort with functional characterization of EZH2 targeting in A549 cells using the catalytic inhibitor EPZ6438 and the EZH2 degrader MS1943. Elevated EZH2 expression was associated with enrichment of cell cycle-related transcriptional pathways. Pharmacological targeting of EZH2 reduced proliferation, migration, stemness-associated features, and sphere-forming capacity, with more pronounced effects observed following EZH2 degradation. Both compounds promoted features consistent with senescence-associated phenotypic remodeling characterized by increased expression of p16 and p21, enhanced β-galactosidase activity, G0/G1 cell cycle arrest, and increased expression of cytokines commonly associated with senescence-related secretory signaling, including IL-6, CCL2, and CXCL8. Conditioned medium from treated tumor cells promoted activation of primary lung fibroblasts, indicating functional paracrine microenvironmental remodeling. Importantly, EZH2 targeting elicited cytostatic responses without induction of apoptosis. Collectively, these findings suggest that EZH2 contributes to regulation of proliferation-associated and senescence-associated phenotypic programs together with stromal signaling in lung adenocarcinoma.

## 1. Introduction

Lung cancer remains the leading cause of cancer-related mortality worldwide and continues to represent a major global health burden despite advances in molecular diagnostics and targeted therapeutic strategies [1,2]. Among its histological subtypes, lung adenocarcinoma (LUAD) is characterized by pronounced genomic and epigenomic heterogeneity that contributes to tumor progression, therapy resistance, and dynamic remodeling of the tumor microenvironment (TME) [3,4,5]. Increasing evidence indicates that chromatin-modifying enzymes coordinate transcriptional plasticity in response to oncogenic signaling and environmental stress conditions within tumor tissues [6,7].

Enhancer of zeste homolog 2 (EZH2), the catalytic subunit of the polycomb repressive complex 2 (PRC2), mediates trimethylation of histone H3 lysine 27 (H3K27me3) and thereby regulates transcriptional silencing of genes involved in proliferation, differentiation, and tumor suppression [6,8,9]. Aberrant EZH2 activity has been implicated in multiple oncogenic processes [10,11,12]. In lung adenocarcinoma, elevated EZH2 expression correlates with aggressive clinical behavior and unfavorable prognosis, supporting its functional relevance as both a prognostic biomarker and therapeutic target [13,14,15]. Consistently, transcriptomic analyses of LUAD cohorts indicate that tumors with high EZH2 expression are enriched in gene programs associated with cell cycle progression, mitotic activity, and DNA replication, suggesting that EZH2 contributes to maintenance of proliferative tumor states [16,17].

Beyond its canonical role in transcriptional repression, EZH2 has emerged as an important regulator of cellular senescence, a state of stable proliferative arrest that can arise from both stress-induced and replicative mechanisms [18,19]. In non-small cell lung cancer, EZH2-mediated repression of CDKN2A-dependent pathways contributes to suppression of senescence programs and preservation of proliferative capacity, indicating that chromatin-level regulation represents an important determinant of senescence escape in tumor cells [20,21].

Accumulating evidence indicates that senescence-associated cytokine signaling may influence tumor–stromal communication and fibroblast activation within the lung cancer microenvironment. However, whether EZH2-dependent senescence-associated signaling directly contributes to functional activation of stromal fibroblasts in lung adenocarcinoma remains insufficiently explored. Clarifying this relationship may provide important insight into how chromatin-level regulation of tumor cell plasticity translates into microenvironmental remodeling processes [22,23,24,25].

In addition to its role in proliferation and senescence regulation, EZH2 contributes to maintenance of stemness-associated transcriptional programs in multiple malignancies, including NSCLC, where it supports tumor cell plasticity and self-renewal capacity [26,27,28,29]. Importantly, EZH2 targeting has also been reported to induce cytostatic responses without triggering overt apoptotic cell death in selected tumor contexts, suggesting that chromatin-level reprogramming may redirect tumor cells toward persistent cytostatic responses rather than cytotoxic elimination [30,31]. Such responses are frequently accompanied by senescence-associated cytokine signaling capable of reshaping tumor–stromal communication networks [32,33].

Therapeutic targeting of EZH2 has therefore emerged as a promising strategy in oncology. Tazemetostat (EPZ6438), a selective inhibitor of EZH2 catalytic activity, has demonstrated clinical efficacy and received regulatory approval for selected malignancies, highlighting the translational relevance of PRC2 inhibition [34,35,36]. However, catalytic inhibition does not fully eliminate non-canonical EZH2 functions that may contribute to tumor cell plasticity and resistance-associated signaling pathways [37,38,39,40]. Recently developed targeted protein degraders, including MS1943, enable proteasomal removal of EZH2 and therefore represent a mechanistically distinct strategy capable of suppressing both enzymatic and scaffold-related functions of this protein [39,41,42,43].

Despite increasing recognition of EZH2 as a key regulator of lung cancer biology, the biological consequences of pharmacological EZH2 degradation remain insufficiently characterized in lung adenocarcinoma models [31,44].

Therefore, the present study investigates the role of EZH2 in the regulation of proliferation, stemness, senescence-associated signaling, and tumor–stromal interactions in lung cancer cells and evaluates the biological effects of catalytic inhibition with EPZ6438 and targeted degradation using MS1943 as complementary epigenetic intervention strategies. By integrating transcriptomic analyses of LUAD cohorts with functional studies in A549 cells, we sought to determine how EZH2 targeting influences proliferation, senescence-associated signaling, and fibroblast activation, and whether targeted EZH2 degradation provides biological advantages over catalytic inhibition. We hypothesized that targeted degradation of EZH2, in contrast to selective catalytic inhibition, may more effectively promote senescence-associated transcriptional remodeling and cytokine signaling consistent with a senescence-associated secretory phenotype-like response, thereby promoting functional activation of lung fibroblasts and reshaping tumor–stromal communication networks.

## 2. Results

### 2.1. EZH2 Overexpression Is Associated with a Proliferative Transcriptional Program in Lung Adenocarcinoma Cohorts

**Figure 1:**
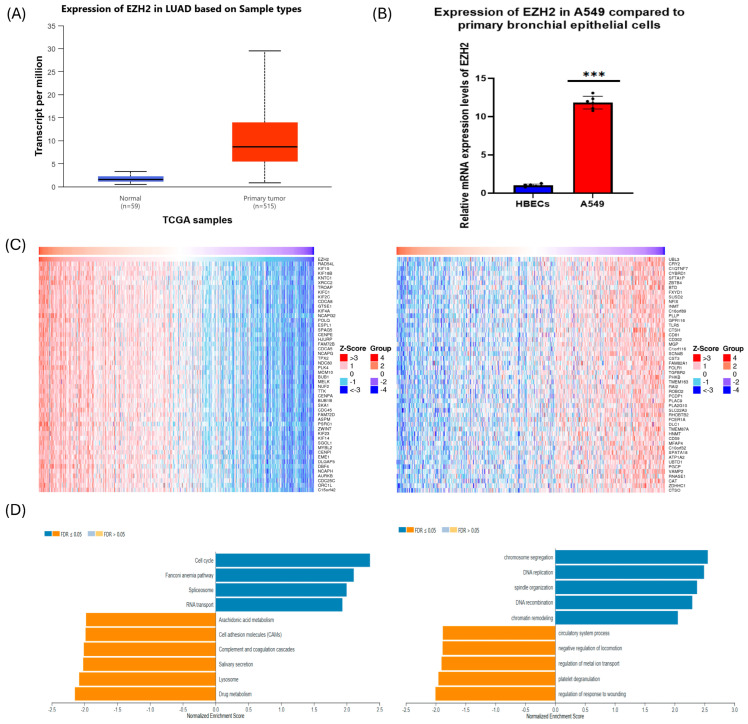
Transcriptomic landscape of EZH2 in lung adenocarcinoma. (**A**) Boxplot of EZH2 mRNA expression in normal lung tissue versus primary tumors from The Cancer Genome Atlas Lung Adenocarcinoma cohort, demonstrating significant upregulation in tumors. (**B**) Relative mRNA expression levels of EZH2 between A549 and HBECs, which served as the non-malignant reference control, determined by RT-qPCR. Data are normalized to HBEC group and presented as mean ± SEM. Statistical significance was assessed relative to control (*** *p* < 0.001). (**C**) Heatmaps of differentially expressed genes stratified by EZH2 expression. Expression values are shown as Z-score-normalized intensities. (**D**) Gene set enrichment analysis (GSEA) of Gene Ontology (biological processes) and KEGG pathways. Pathways enriched in EZH2-high samples are indicated by positive normalized enrichment scores (NES; FDR < 0.05), whereas pathways enriched in EZH2-low samples are indicated by negative NES (FDR < 0.05).

Initially, we conducted a bioinformatic analysis of The Cancer Genome Atlas Lung Adenocarcinoma (TCGA-LUAD) cohort to assess the expression and functional relevance of EZH2 in adenocarcinoma samples. Analysis of EZH2 mRNA levels demonstrated significant upregulation in primary tumors (n = 515) compared with normal lung tissue (n = 59), indicating overexpression of EZH2 in cancer cells. Additionally, we have performed qRT-PCR analysis demonstrating significantly elevated EZH2 mRNA expression in A549 cells compared to primary human bronchial epithelial cells (HBECs), further supporting EZH2 upregulation in the context of lung malignancy. Unsupervised clustering of differentially expressed genes revealed that EZH2-high tumors are characterized by coordinated upregulation of genes associated with cell cycle progression, mitotic activity, and DNA replication. Gene Set Enrichment Analysis (GSEA), encompassing Gene Ontology (biological processes) and KEGG pathways, demonstrated that high EZH2 expression is associated with enrichment of pathways related to cell cycle progression, including DNA replication, chromosome segregation, and RNA transport. In contrast, EZH2 expression exhibited an inverse correlation with metabolic and microenvironmental processes (Figure 1).

These findings prompted the hypothesis that EZH2 may contribute to regulation of proliferation and cell cycle progression in lung adenocarcinoma, as well as potentially influencing tumor metabolism and microenvironmental signaling. These processes may contribute to accelerated tumor progression and development of an aggressive disease phenotype.

### 2.2. EZH2 Targeting Does Not Induce Overt Cytotoxicity in A549 Cells and Normal Lung Cells

**Figure 2:**
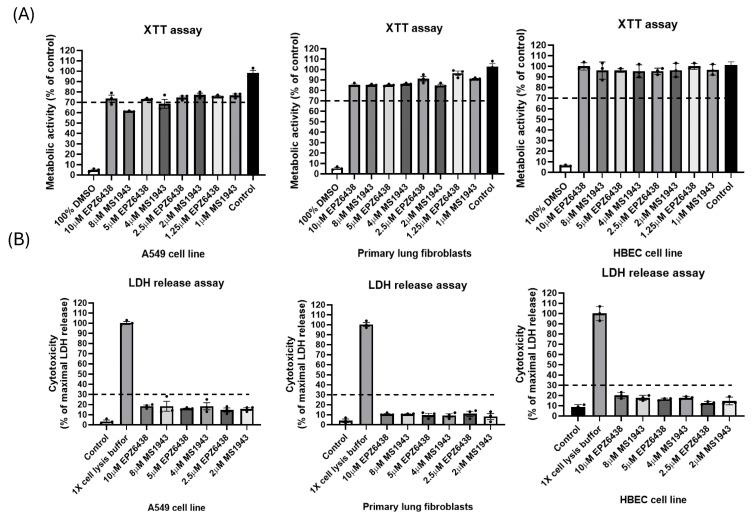
Assessment of cell viability and cytotoxicity following EZH2 targeting in A549 cells and normal lung cells. (**A**) XTT assay assessing metabolic activity in A549 cells, primary lung fibroblasts and HBEC cells line across a range of EPZ6438 and MS1943 concentrations. Data are presented as percentage of control (mean ± SEM). (**B**) LDH release assay measuring cytotoxicity in A549 cells, primary lung fibroblasts and HBEC cell line. Data are presented as percentage of maximal LDH release (mean ± SD).

The XTT assay demonstrated largely preserved metabolic activity across most concentrations in A549 cells, primary lung fibroblasts and HBEC cell line, with a biologically relevant decrease (>30% relative to control viability) observed only in A549 cells at the highest doses of the tested compounds (Figure 2A). In parallel, LDH release remained minimal and comparable to control conditions (Figure 2B), indicating maintenance of membrane integrity and absence of overt cytotoxicity. Together, these findings indicate that EPZ6438 and MS1943 did not induce substantial membrane-damaging cytotoxicity under the experimental conditions applied.

### 2.3. Pharmacological Targeting of EZH2 Suppresses Proliferation, Migration, Mesenchymal Phenotype, and Stemness in the A549 Lung Adenocarcinoma Cell Line

#### 2.3.1. Effects of EZH2 Inhibition on Proliferation of A549 Cells

**Figure 3:**
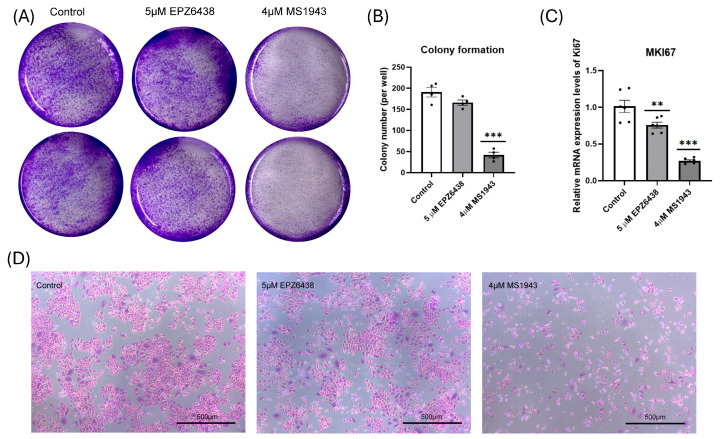
EZH2 inhibition suppresses colony formation and MKI67 expression in A549 cells. (**A**) Representative images of crystal violet-stained colony formation assays under control conditions and following treatment with EPZ6438 (5 μM) or MS1943 (4 μM). (**B**) Quantification of colony number per well. Data are presented as mean ± SEM. Statistical significance was assessed relative to control (*** *p* < 0.001). (**C**) Relative mRNA expression levels of MKI67 determined by RT-qPCR following treatment. Data are normalized to control and presented as mean ± SEM. Statistical significance was assessed relative to control (** *p* < 0.01, ***p < 0.001). (**D**) Representative phase-contrast images showing cell morphology and density across treatment conditions.

Unless otherwise indicated, molecular and phenotypic analyses were performed after 72 h of treatment, a time point selected to capture early senescence-associated phenotypic changes. Colony formation assays were conducted over a 10-day treatment period. To address the role of EZH2 in lung adenocarcinoma biology, we treated the A549 cell line with the FDA-approved EZH2 inhibitor EPZ6438 (tazemetostat) and the recently developed EZH2 degrader MS1943. Following 10 days of continuous treatment, colony formation assays demonstrated a marked reduction in both colony size and density following treatment with MS1943 (Figure 3A,B,D). Morphological assessment corroborated these findings, as treated cells exhibited decreased confluency and increased cell dispersion in the MS1943-treated group (Figure 3D). Consistently, analysis of MKI67 expression revealed significant downregulation at the mRNA level following EZH2 targeting (Figure 3C), further supporting reduced proliferative activity. Although EPZ6438 significantly reduced MKI67 expression at the transcript level, this effect was not accompanied by a statistically significant decrease in colony formation, suggesting that catalytic inhibition alone may be insufficient to suppress clonogenic growth in A549 cells.

#### 2.3.2. Effects of EZH2 Inhibition on A549 Cells Migration

**Figure 4:**
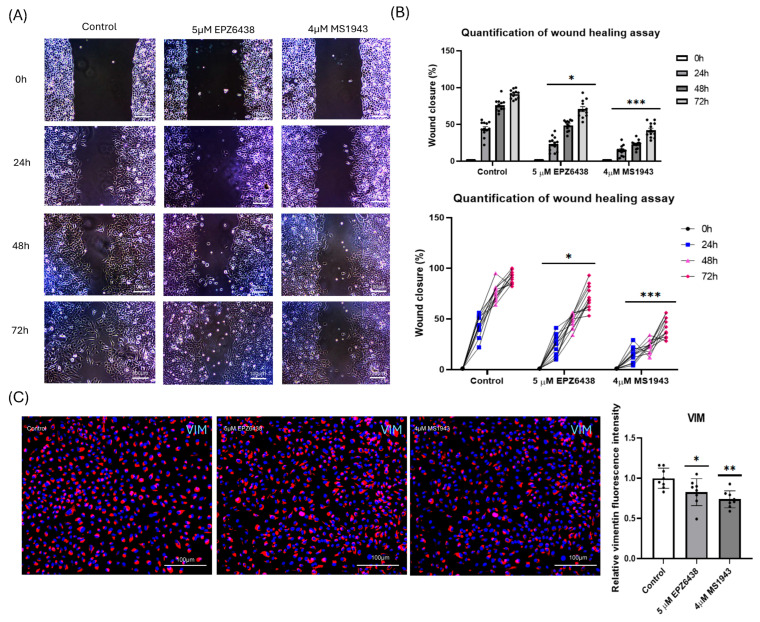
EZH2 inhibition impairs migration and reduces mesenchymal marker expression in A549 cells. (**A**) Representative images of wound healing assays at 0 h, 24 h, 48 h, and 72 h under control conditions and following treatment with EPZ6438 (5 μM) or MS1943 (4 μM). (**B**) Quantification of wound closure (%) over time. Upper panel: bar graph showing mean ± SEM; lower panel: individual replicate trajectories. Statistical significance was assessed relative to control (* *p* < 0.05, *** *p* < 0.001). (**C**) Representative immunofluorescence images and relative fluorescence intensity quantification of vimentin (red) with nuclear counterstaining (DAPI, blue) across treatment conditions. Statistical significance was assessed relative to control (* *p* < 0.05, ** *p* < 0.01).

Wound healing assays demonstrated time-dependent closure of the scratch area in control cells, whereas treatment with both EPZ6438 and MS1943 significantly delayed wound closure (Figure 4A). Quantitative analysis confirmed a significant reduction in migration in both treatment groups compared with control, with the strongest inhibitory effect observed following EZH2 degradation by MS1943 (Figure 4B). Quantitative immunofluorescence analysis of vimentin revealed reduced expression following EZH2 targeting (Figure 4C), suggesting attenuation of a mesenchymal-like phenotype associated with reduced migratory plasticity.

#### 2.3.3. Effects of EZH2 Inhibition on A549 Spheroids Formation and Stemness Marker Expression

**Figure 5:**
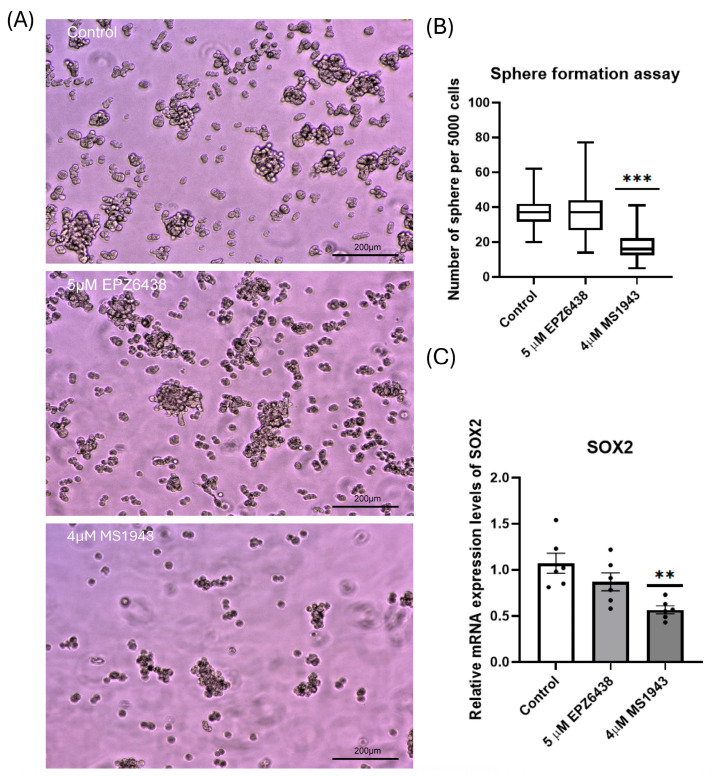
EZH2 targeting impairs sphere formation and reduces stemness marker expression in A549 cells. (**A**) Representative images of sphere formation assays under control conditions and following treatment with EPZ6438 (5 μM) or MS1943 (4 μM). (**B**) Quantification of sphere number per 5000 seeded cells. Data are presented as box plots with median and interquartile range. Statistical significance was assessed relative to control (*** *p* < 0.001). (**C)** Relative mRNA expression levels of SOX2 determined by RT-qPCR following treatment. Data are normalized to control and presented as mean ± SEM. Statistical significance was assessed relative to control (** *p* < 0.01).

Sphere formation assays demonstrated a decrease in both the number and size of spheres following treatment with MS1943 (Figure 5A). Quantitative analysis confirmed a significant reduction in sphere-forming capacity in the MS1943-treated group compared with control (Figure 5B), indicating impaired self-renewal potential. Consistently, expression analysis of SOX2, a key stemness marker, demonstrated significant downregulation following EZH2 degradation (Figure 5C). In contrast, EPZ6438 treatment did not produce a statistically significant reduction in sphere-forming capacity or SOX2 expression under the experimental conditions applied, suggesting that maintenance of stemness-associated transcriptional programs may depend not only on canonical H3K27me3-mediated repression but also on non-canonical EZH2 functions that are more effectively disrupted by targeted protein degradation.

### 2.4. EZH2 Targeting Promotes Senescence-Associated Phenotypic Remodeling, Cell Cycle Arrest, and Cytokine Signaling in A549 Cell, Promoting Cancer-Associated Fibroblast Activation

#### 2.4.1. Targeting EZH2 Promotes Features Consistent with a Senescence-like Phenotype in A549 Cells

**Figure 6:**
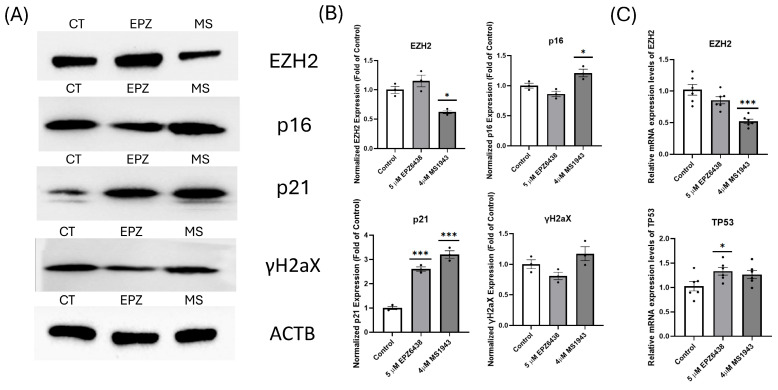
EZH2 expression is inversely associated with expression of key senescence markers in A549 cells at the protein level. (**A**) Representative Western blot analysis of EZH2, p16, p21, and γH2AX in control (CT) cells and cells treated with EPZ6438 (5 μM) or MS1943 (4 μM). ACTB served as a loading control. (**B**) Quantification of protein expression normalized to control. Data are presented as mean ± SEM. Statistical significance was assessed relative to control (* *p* < 0.05, *** *p* < 0.001). (**C**) Relative mRNA expression levels of EZH2 and TP53 determined by RT-qPCR. Data are normalized to control and presented as mean ± SEM. Statistical significance was assessed relative to control (* *p* < 0.05, *** *p* < 0.001).

Western blot analysis demonstrated reduced EZH2 protein levels following MS1943 treatment, as indicated by weaker EZH2 bands compared with control and EPZ6438-treated cells. This was accompanied by a marked increase in the intensity of p16 and p21 bands, consistent with acquisition of features associated with a senescence-like cytostatic phenotype (Figure 6A,B). Densitometric analysis confirmed a significant decrease in EZH2 protein levels alongside upregulation of p16 and p21 in the MS1943-treated group, whereas in EPZ6438-treated cells only p21 expression was significantly increased (Figure 6B). In contrast, γH2AX and p53 exhibited only modest changes in band intensity, suggesting limited activation of DNA damage signaling and p53-dependent pathways. These observations are consistent with early-stage senescence-associated changes detected after 72 h of treatment.

At the transcriptional level, EZH2 mRNA expression was significantly reduced following MS1943 treatment, whereas TP53 expression showed a moderate increase upon EPZ6438 treatment (Figure 6C).

**Figure 7:**
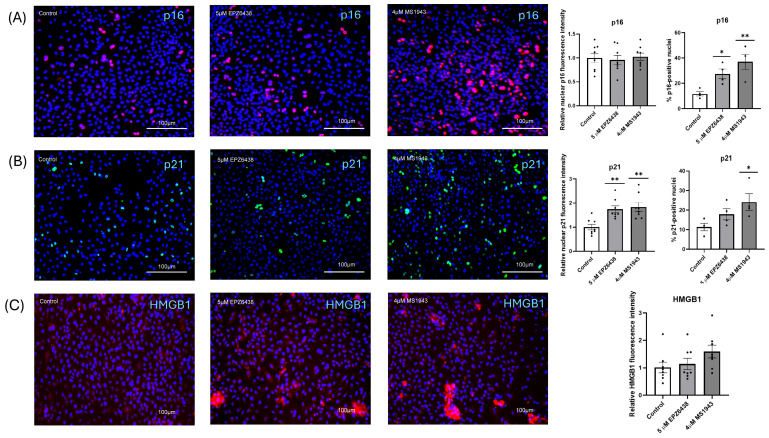
EZH2 targeting promotes features consistent with senescence-associated phenotypic remodeling in A549 cells. (**A**) Representative immunofluorescence images, quantitative analysis of relative fluorescence intensity, and percentage of p16-positive cells demonstrating p16 staining (Texas Red, red) with nuclear counterstaining using DAPI (blue). (**B**) Representative immunofluorescence images, quantitative analysis of relative fluorescence intensity, and percentage of p21-positive cells demonstrating p21 staining (FITC, green) with nuclear counterstaining using DAPI (blue). (**C**) Representative immunofluorescence images and relative fluorescence intensity quantification of HMGB1 (Texas Red, red) with DAPI nuclear staining (DAPI, blue). Data are normalized to control and presented as mean ± SEM. Statistical significance was assessed relative to control (* *p* < 0.05, ** *p* < 0.01).

Quantitative immunofluorescence analysis further supported the presence of features consistent with a senescence-associated phenotype. Both p16 and p21 displayed increased staining intensity and a higher proportion of positive cells following EZH2 targeting, most prominently in the MS1943-treated group (Figure 7A,B). Concurrently, HMGB1 demonstrated reduced intracellular localization accompanied by increased extracellular signal (Figure 7C), consistent with acquisition of a senescence-associated chromatin remodeling phenotype.

#### 2.4.2. EZH2 Targeting Induces G1 Cell Cycle Arrest and Increases β-Galactosidase Activity in A549 Cells

**Figure 8:**
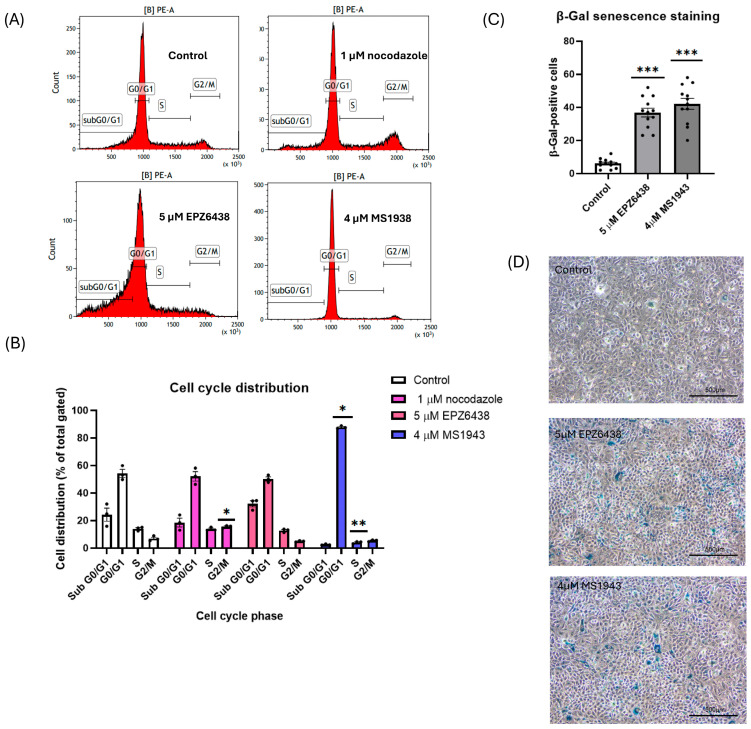
EZH2 targeting induces G1 arrest and promotes senescence-associated phenotypic changes in A549 cells. (**A**) Representative flow cytometry histograms (PI staining) showing cell cycle distribution in control cells and following treatment with nocodazole (1 μM), EPZ6438 (5 μM), or MS1943 (4 μM). Cell cycle phases (subG0/G1, G0/G1, S, G2/M) are indicated. (**B**) Quantification of cell cycle distribution (% of total gated cells) across conditions. Data are presented as mean ± SEM. Statistical significance was assessed relative to control (* *p* < 0.05, ** *p* < 0.01). (**C**) Quantification of β-galactosidase-positive cells following treatment. Data are presented as mean ± SEM. Statistical significance was assessed relative to control (*** *p* < 0.001). (**D**) Representative images of β-galactosidase staining in control and treated cells.

Flow cytometric cell cycle analysis demonstrated that treatment of A549 cells with the EZH2 degrader MS1943 induced pronounced accumulation of cells in the G0/G1 phase, accompanied by a reduction in S-phase and G2/M populations, consistent with acquisition of a cytostatic G1-arrested phenotype commonly associated with senescence-like cellular remodeling (Figure 8A,B) [45]. In contrast, EPZ6438 induced more modest changes, with a partial increase in the subG0/G1 fraction and only minor alterations in S-phase distribution.

Β-galactosidase (β-Gal) staining demonstrated a significant increase in the proportion of β-Gal-positive cells following EZH2 targeting, with the strongest effect observed in the MS1943-treated group (Figure 8C,D). Morphologically, treated cells displayed enlarged and flattened phenotypes commonly associated with senescence-like phenotypic remodeling [46].

#### 2.4.3. EZH2 Targeting Increases Expression of Cytokines Associated with Senescence-Related Secretory Signaling and Promotes Cancer-Associated Fibroblast Activation in Primary Murine Lung Fibroblasts

**Figure 9:**
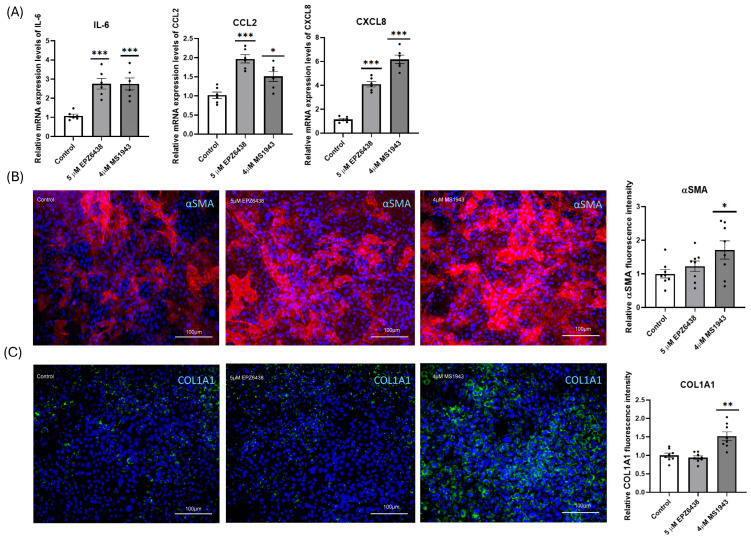
EZH2 targeting induces SASP and promotes cancer-associated fibroblast activation. (**A**) Relative mRNA expression levels of IL6, CCL2, and CXCL8 determined by RT-qPCR following treatment with EPZ6438 (5 μM) or MS1943 (4 μM). Data are normalized to control and presented as mean ± SEM. Statistical significance was assessed relative to control (* *p* < 0.05, *** *p* < 0.001). (**B**) Representative immunofluorescence images and relative fluorescence intensity quantification of αSMA (Texas Red, red) with DAPI nuclear staining (DAPI, blue), indicating fibroblast activation. (**C**) Representative immunofluorescence images of COL1A1 and relative fluorescence intensity quantification (FITC, green) with DAPI nuclear staining (DAPI, blue), indicating extracellular matrix remodeling. Data are normalized to control and presented as mean ± SEM. Statistical significance was assessed relative to control (* *p* < 0.05, ** *p* < 0.01).

RT-qPCR analysis of A549 cells demonstrated significant upregulation of pro-inflammatory cytokines commonly associated with the senescence-associated secretory phenotype-like response (Figure 9A). Subsequent exposure of murine primary lung fibroblasts to conditioned medium derived from EPZ6438- and MS1943-treated A549 cells resulted in enhanced expression of cancer-associated fibroblast (CAF) markers. Immunofluorescence analysis revealed increased αSMA and COL1A1 staining, with the most pronounced effects observed in fibroblasts exposed to conditioned medium from MS1943-treated cells (Figure 9B,C).

### 2.5. EZH2 Targeting Elicits Cellular Stress Without Triggering Cytotoxicity or Apoptosis in A549 Cells

**Figure 10:**
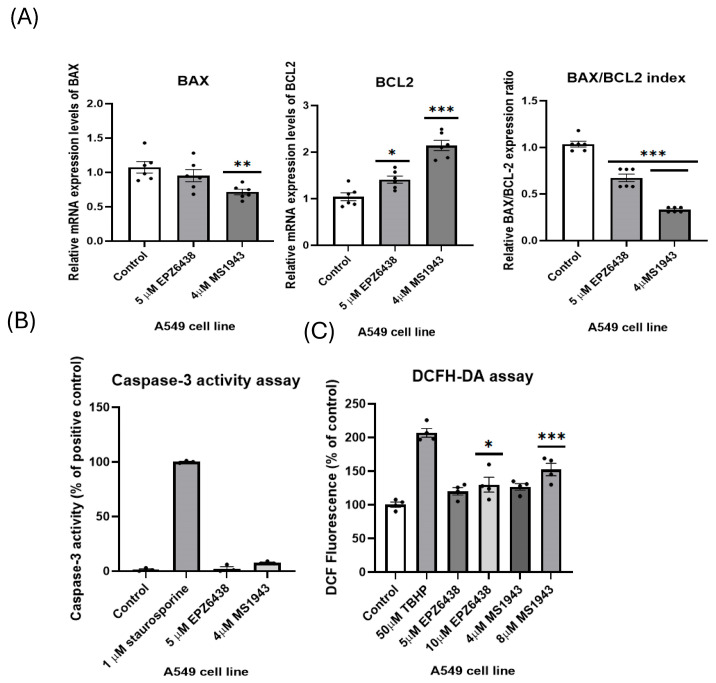
EZH2 targeting induces oxidative stress without activating apoptotic pathways. (**A**) Relative mRNA expression levels of BAX, BCL2, and BAX/BCL2 ratio determined by RT-qPCR in A549 cells. Data are normalized to control and presented as mean ± SEM. Statistical significance was assessed relative to control (* *p* < 0.05, ** *p* < 0.01, *** *p* < 0.001). (**B**) Caspase-3 activity assay in A549 cells following treatment, with staurosporine as a positive control. Data are presented as percentage of positive control (mean ± SEM). (**C**) DCFH-DA assay measuring intracellular ROS levels in A549 cells following treatment with EPZ6438 and MS1943. Data are presented as percentage of control (mean ± SEM). Statistical significance was assessed relative to control (* *p* < 0.05, *** *p* < 0.001).

Analysis of apoptosis-related markers in A549 cells revealed decreased BAX expression and increased BCL2 levels following EZH2 targeting, resulting in a reduced BAX/BCL2 ratio (Figure 10A). These changes are consistent with suppression of mitochondrial apoptotic signaling. In agreement with these findings, caspase-3 activity remained low following treatment with EPZ6438 and MS1943, whereas marked activation was observed in staurosporine-treated cells used as a positive control (Figure 10B). Together, these results indicate that pharmacological targeting of EZH2 does not activate canonical apoptotic pathways in A549 cells.

The DCFH-DA assay revealed significantly increased intracellular ROS levels following EZH2 targeting (Figure 10C). However, this effect was observed only at the highest concentrations of EPZ6438 and MS1943, suggesting induction of a secondary cellular stress response rather than a primary mechanism underlying the observed phenotypic changes.

## 3. Discussion

Our data demonstrate that pharmacological targeting of EZH2 promotes a cytostatic response associated with features of early senescence-like phenotypic remodeling in A549 lung adenocarcinoma cells, as reflected by reduced colony formation, decreased MKI67 expression, altered cell cycle distribution, increased β-galactosidase activity, and induction of cytokine signaling associated with a senescence-related secretory phenotype-like response. These findings are consistent with previous reports demonstrating impaired proliferation following EZH2 inhibition across multiple malignancies, including NSCLC [47,48].

EZH2 targeting triggered features consistent with a senescence-associated phenotype, including increased expression of pro-inflammatory cytokines commonly associated with senescence-related secretory signaling, namely CXCL8, CCL2, and IL-6, upregulation of the cell cycle regulators p21 and p16, enhanced β-galactosidase activity, and accumulation of cells in the G0/G1 phase with a corresponding reduction in S and G2/M populations. Together, these observations suggest that EZH2 may contribute to chromatin-level regulation of pathways associated with senescence escape in lung adenocarcinoma cells.

Our findings align with previous studies showing that EZH2 depletion promotes senescence-associated phenotypic remodeling characterized by increased β-galactosidase activity, enhanced expression of cytokines associated with senescence-related secretory signaling, and persistent cytostatic growth arrest [49]. Although these studies demonstrated induction of senescence-associated phenotypes following EZH2 depletion, they did not specifically address tumor–stromal communication in lung adenocarcinoma models. In NSCLC models, EZH2 silencing has also been linked to G1 phase accumulation and reduced S-phase entry [50].

Simultaneously, we observed that EZH2 targeting did not trigger overt cytotoxicity or induce apoptotic cell death in A549 cells, as demonstrated by unchanged caspase-3 activity, preserved metabolic activity, and minimal LDH release. This was accompanied by a shift in BCL-2 family gene expression, with a reduced BAX/BCL2 ratio, suggesting a bias toward cell survival rather than apoptosis. These findings indicate that EZH2 targeting primarily promotes a cytostatic response rather than activation of classical apoptotic pathways. These observations contrast with previous studies reporting pro-apoptotic effects of EZH2 inhibition in selected NSCLC and other cancer [51,52]. Such discrepancies likely reflect differences in molecular background and epigenetic dependencies across tumor types [53]. Importantly, in our system, the dominant outcome of EZH2 targeting appears to favor a persistent cytostatic state associated with features of a senescence-like phenotype.

To evaluate distinct EZH2-targeting strategies, we compared the effects of the FDA-approved catalytic inhibitor EPZ6438, also known as tazemetostat, and the hydrophobic-tagged degrader MS1943 [54,55].

Notably, MS1943 induced more pronounced senescence-associated phenotypic changes than EPZ6438, as indicated by higher expression of senescence-associated markers and substantially increased β-galactosidase activity. Flow cytometric analysis further revealed a marked difference between the two targeting strategies, as only MS1943 induced pronounced G0/G1 arrest with a corresponding reduction in S and G2/M populations. Consistently, MS1943 treatment resulted in stronger suppression of proliferative and migratory capacity than EPZ6438.

The discrepancy between reduced MKI67 expression and preserved clonogenic growth following EPZ6438 treatment suggests that catalytic inhibition of EZH2 may partially attenuate proliferative signaling without fully suppressing the long-term reproductive capacity of A549 cells. In addition, a pronounced decrease in SOX2 expression and impaired sphere-forming ability were observed exclusively following EZH2 degradation. These findings suggest that targeted degradation of EZH2 represents a more comprehensive epigenetic intervention strategy than catalytic inhibition alone in lung adenocarcinoma cells. The enhanced activity of MS1943 may be attributed to its ability to disrupt both enzymatic and non-canonical scaffold functions of EZH2, which have been increasingly recognized as important determinants of tumor cell plasticity and therapeutic resistance [31,37,38,54].

The senescence-associated phenotypic remodeling observed following EZH2 targeting indicates a cytostatic effect that may contribute to suppression of tumor growth. Senescent tumor cells can also actively remodel their microenvironment through secretion of pro-inflammatory cytokines and matrix-modifying factors, including IL-6, CXCL8, CCL2, and metalloproteinases, thereby influencing immune signaling and stromal activation [56,57]. Cancer-associated fibroblasts, which represent a major stromal component of lung tumors, participate in reciprocal signaling interactions with tumor cells and contribute to extracellular matrix remodeling, immune suppression, and metastatic dissemination [56,58,59].

These observations support the concept that senescence-associated cytokine signaling may represent an important mechanism linking tumor-intrinsic chromatin regulation with microenvironmental remodeling processes in NSCLC. However, stable proliferative arrest cellular senescence represents a complex biological program with potentially dual roles in cancer progression [60,61].

Consistent with this concept, our data demonstrate increased expression of cytokines associated with senescence-related secretory signaling together with enhanced expression of cancer-associated fibroblast markers following exposure to conditioned medium derived from EZH2-targeted tumor cells. These findings support a role for EZH2-dependent chromatin regulation in coordinating tumor–stromal signaling interactions. They also indicate that the biological consequences of EZH2 targeting extend beyond tumor cell-autonomous effects and may include paracrine remodeling of the surrounding stromal compartment [62].

Given that EZH2 has emerged as an important therapeutic target in drug-resistant NSCLC [63], these observations raise important considerations regarding the broader biological consequences of EZH2-targeting strategies. In this context, targeted EZH2 degradation may represent a promising component of combination-oriented therapeutic approaches, including strategies involving EGFR tyrosine kinase inhibitors, senolytic agents aimed at eliminating therapy-induced senescent cells, or immunomodulatory interventions designed to reshape senescence-associated cytokine-mediated tumor-microenvironment interactions. Nevertheless, the impact of EZH2 inhibition in cancer therapy appears to be highly context-dependent, particularly in light of its involvement in cellular senescence, which may exert both tumor-suppressive and tumor-promoting effects [64].

It should be noted that the 72 h time point primarily captures early stages of senescence-associated phenotypic remodeling. At this stage, certain features may partially overlap with acute stress or inflammatory responses. Therefore, while the combined phenotypic and molecular data support induction of a senescence-like state, further time-course studies will be required to confirm the establishment of long-term senescence and a fully developed senescence-associated secretory phenotype-like response.

An additional limitation of the present study is the use of a single lung adenocarcinoma cell line. Given the substantial molecular and epigenetic heterogeneity of LUAD, together with the context-dependent nature of senescence-associated signaling, the observed phenotypic responses may not be universally conserved across all NSCLC models. Therefore, future studies employing genetically distinct LUAD and NSCLC cell lines will be necessary to determine the broader applicability of EZH2-targeting-induced senescence-associated phenotypic remodeling and tumor–stromal signaling responses.

A further limitation is the use of murine lung fibroblasts in co-culture experiments with human A549 lung adenocarcinoma cells. Although this approach enabled proof-of-concept evaluation of stromal activation induced by EZH2-targeted tumor cells, species-specific differences in cytokine signaling and fibroblast responsiveness may influence the observed interactions. Additional experiments performed using normal human primary bronchial/tracheal epithelial cells demonstrated that EZH2-targeting compounds did not induce overt cytotoxicity in non-malignant human pulmonary cells, thereby supporting the selectivity of the experimental conditions. Nevertheless, future studies employing primary human lung fibroblasts or fully human co-culture systems will be required to further validate tumor–stromal interactions associated with EZH2 targeting.

**Figure 11:**
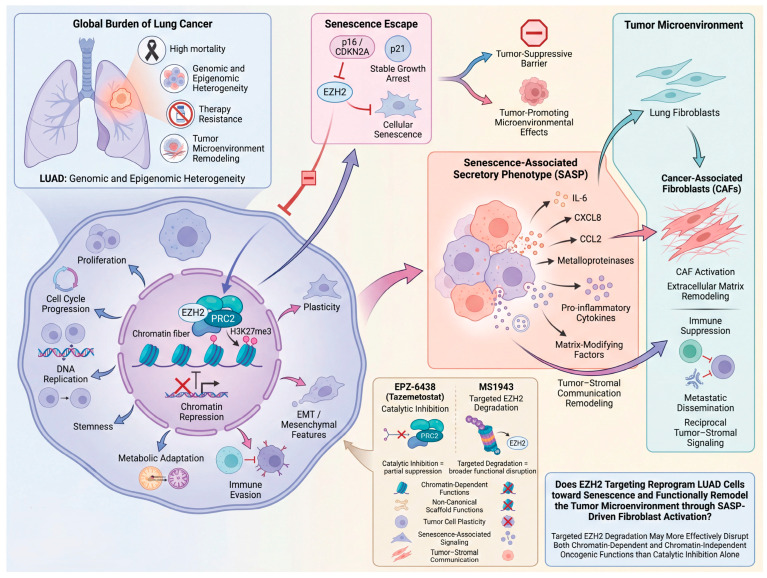
Conceptual model of EZH2-dependent regulation of senescence-associated phenotypic remodeling and fibroblast activation in lung adenocarcinoma. EZH2-mediated chromatin repression supports proliferative tumor states and limits pathways associated with senescence-related signaling in LUAD cells. Pharmacological EZH2 targeting promotes cytokine signaling consistent with a senescence-associated secretory phenotype-like response, which may contribute to cancer-associated fibroblast activation and tumor–stromal communication. Compared with catalytic inhibition by EPZ6438, targeted EZH2 degradation by MS1943 enables broader disruption of chromatin-dependent and chromatin-independent EZH2 functions and may more effectively remodel microenvironmental signaling pathways.

The proposed relationship between EZH2-dependent chromatin regulation, senescence-associated signaling, and fibroblast activation identified in the present study is summarized in the conceptual model shown in Figure 11.

Taken together, our findings suggest that EZH2 may function as an important epigenetic regulator coordinating proliferation, stemness-associated programs, and senescence-related tumor–stromal communication in lung adenocarcinoma cells. Within this framework, pharmacological degradation of EZH2 promotes a cytostatic response associated with features of senescence-associated phenotypic remodeling, accompanied by activation of cytokine signaling consistent with a senescence-associated secretory phenotype-like response and paracrine activation of fibroblasts without induction of apoptotic cell death. These observations indicate that targeted EZH2 degradation represents a mechanistically distinct intervention compared with catalytic inhibition alone and may provide a promising strategy for epigenetic reprogramming of tumor cell plasticity in NSCLC. However, validation in additional experimental models will be required to determine the broader translational relevance of this mechanism.

## 4. Materials and Methods

### 4.1. Bioinformatics

Publicly available TCGA-LUAD transcriptomic data were analyzed to evaluate EZH2 expression and its functional associations. Differential expression between normal and tumor tissues was assessed using the UALCAN platform [65,66]. Correlation analysis, identification of EZH2-associated genes and functional enrichment analysis were performed using LinkedOmics [67]. The top positively and negatively correlated genes were visualized as heatmaps based on Z-score-normalized expression. Functional enrichment analysis (GO biological processes and KEGG pathways) was reported as normalized enrichment scores (NES), with significance defined as FDR < 0.05.

### 4.2. Cell Culture

A549 human lung adenocarcinoma cells (ATCC CCL-185™) were obtained from the American Type Culture Collection (ATCC, Manassas, VA, USA) and cultured in high-glucose DMEM supplemented with 10% fetal bovine serum (FBS) and 1% penicillin-streptomycin (Capricorn Scientific GmbH, Ebsdorfergrund, Germany).

Primary lung fibroblasts were isolated from P3 C57BL/6 mouse pups as previously described, with minor modifications [68]. Briefly, excised lungs were rinsed with ice-cold phenol red-free HBSS (Capricorn Scientific GmbH, Ebsdorfergrund, Germany) and finely minced into approximately 1 mm fragments using sterile scissors. Lung samples were then subjected to enzymatic digestion with 0.25% trypsin under continuous agitation (180 rpm) for approximately 30 min at 37 °C in a CO_2_-free incubator. The resulting cell suspension was filtered through a 70 μm cell strainer to remove debris. Cells were pelleted by centrifugation at 300× *g* for 5 min and resuspended in DMEM supplemented with 10% FBS and 1% penicillin-streptomycin. The suspension was plated onto culture dishes, and after 6 h, non-adherent and weakly attached cells were removed by complete medium replacement. The remaining adherent fibroblasts were maintained at 37 °C in a humidified atmosphere containing 5% CO_2_.

Primary Bronchial/Tracheal Epithelial Cells; Normal, Human (HBEC cell line, PCS-300-010^™^) were obtained from the American Type Culture Collection (ATCC, Manassas, VA, USA) and cultured in collagen I-coated 48-well plate in complete growth media, consisting Airway Epithelial Cell Basal Medium supplemented with Bronchial Epithelial Cell Growth Kit.

For co-culture experiments, A549 cells were seeded onto PET membrane inserts (0.4 μm pore size; SPLInsert™, SPL Life Sciences, Pocheon-si, Republic of Korea) and allowed to attach overnight. Inserts were then transferred into 6-well plates containing primary lung fibroblasts cultured in the lower chamber. A549 cells in the upper compartment were treated with the indicated compounds (EPZ6438 or MS1943) or left untreated as controls for 72 h.

### 4.3. Chemical Treatment

The selective inhibitor of EZH2 histone methyltransferase activity, EPZ6438 (Cat. No. T2557; TargetMol, Boston, MA, USA), and the first-in-class PROTAC-like EZH2 degrader MS1943 (Cat. No. T13780; TargetMol, Boston, MA, USA) were dissolved in DMSO, aliquoted, and stored at −80 °C. A549 cells were treated with the indicated concentrations of each compound, with the final DMSO concentration maintained below 0.1%. Unless otherwise stated, cells were treated for 72 h prior to sample collection. The concentrations of EPZ6438 (5 µM) and MS1943 (4 µM) were selected based on previously published studies together with preliminary dose-response experiments performed in A549 cells [55,69,70,71,72]. These concentrations produced reproducible biological effects while avoiding overt cytotoxicity and apoptosis. Following treatment, cells were subjected to analyses of proliferation, migration, sphere formation, senescence-associated phenotypic remodeling, oxidative stress responses, apoptosis-related signaling, and tumor–stromal interaction assays.

### 4.4. XTT Assay

Cell metabolic activity was assessed using the CyQUANT™ XTT Cell Viability Assay (Thermo Fisher Scientific, Waltham, MA, USA) according to the manufacturer’s instructions. A549 cells were seeded at a density of 1 × 10^4^ cells per well, whereas primary lung fibroblasts and HBECs were seeded at a density of 2 × 10^4^ cells per well in 96-well plates and allowed to attach overnight. Cells were then treated with the indicated compound concentrations for 48 h. At the endpoint, the medium volume was adjusted to 100 μL, and 70 μL of XTT reagent mixed with the Electron Coupling Reagent was added to each well and incubated for 2 h at 37 °C to allow metabolic conversion of XTT into a soluble formazan product. Absorbance was measured spectrophotometrically according to the manufacturer’s instructions. Cell viability was expressed as a percentage relative to untreated control cells.

### 4.5. LDH Release Assay

Cell membrane integrity was assessed using the Pierce™ LDH Cytotoxicity Assay Kit (Thermo Fisher Scientific, Waltham, MA, USA) according to the manufacturer’s instructions. A549 cells, primary lung fibroblasts and HBEC cells were seeded at densities of 1 × 10^4^ and 2 × 10^4^ cells per well, respectively, in 96-well plates and allowed to attach overnight. Cells were then treated with the indicated compound concentrations for 48 h. At the endpoint, 50 μL of culture supernatants was transferred to a new plate, and 50 μL of LDH reaction mixture was added to each well and incubated for 30 min at room temperature, protected from light. Absorbance was measured using a microplate spectrophotometer according to the manufacturer’s protocol. Cytotoxicity was calculated by normalizing LDH release in treated samples to untreated controls (spontaneous release) and expressed as a percentage of maximal LDH release.

### 4.6. Colony Formation Assay

A549 cells were seeded at a density of 1 × 10^3^ cells per well in 12-well plates and cultured for 10 days. Cells were treated with the indicated inhibitors, and fresh DMEM supplemented with 10% FBS containing the compounds was replaced every 3 days. At the endpoint, cells were fixed with 4% buffered paraformaldehyde (PFA) and stained with 0.05% crystal violet for 20 min. Wells were gently washed to remove excess dye and air-dried. Colonies were counted using ImageJ software version 1.54g (National Institutes of Health, Bethesda, MD, USA), and representative images were acquired using an MW50 inverted microscope (OPTA-TECH, Warsaw, Poland). Colony formation was quantified according to standard clonogenic assay methodology, in which a colony is defined as a cluster of at least 50 cells.

### 4.7. Wound Healing Assay

Cell migration was evaluated using Culture-Inserts (ibidi GmbH, Gräfelfing, Germany) placed in 12-well plates. A549 cells were seeded at a density of 3 × 10^4^ cells per chamber and allowed to reach full confluence overnight. Inserts were then removed to create a defined cell-free gap, and cells were gently washed to remove debris. Cells were subsequently incubated in DMEM containing 1% FBS with the indicated treatments. Images were acquired at 0, 24, 48, and 72 h using an MW50 inverted microscope (OPTA-TECH, Warsaw, Poland). Wound closure was quantified using ImageJ software (National Institutes of Health, Bethesda, MD, USA) by measuring the wound area at each time point and expressing it as a percentage of the initial wound area.

### 4.8. Sphere Formation Assay

Sphere formation was performed as previously described with minor adjustments [73]. Briefly, 24-well plates were coated with BIOFLOAT™ FLEX Coating Solution (faCellitate GmbH, Mannheim, Germany) according to the manufacturer’s instructions to prevent cell attachment. A549 cells were seeded at a density of 5 × 10^3^ cells per well in serum-free DMEM/F12 medium supplemented with 1% N2 supplement (Capricorn Scientific GmbH, Ebsdorfergrund, Germany), EGF (20 ng/mL), and FGFb (20 ng/mL) (PeproTech, Rocky Hill, NJ, USA). Cells were treated with the indicated compounds and maintained under non-adherent conditions for 9 days, with fresh medium containing growth factors and inhibitors added every 3 days. Sphere formation was imaged using an MW50 inverted microscope (OPTA-TECH, Warsaw, Poland) and quantification was performed using ImageJ software (National Institutes of Health, Bethesda, MD, USA) with a sphere size threshold of ≥50 μm in diameter. The number of spheres per well was calculated and normalized to the number of seeded cells.

### 4.9. RNA Isolation and RT-qPCR

Total RNA from untreated HBEC cells and A549 cells after indicated treatments and 72 h incubation time was isolated using the RNAqueous™ Total RNA Isolation Kit (Invitrogen, Thermo Fisher Scientific, Waltham, MA, USA) according to the manufacturer’s instructions. Complementary DNA (cDNA) was synthesized using the High-Capacity cDNA Reverse Transcription Kit (Applied Biosystems, Thermo Fisher Scientific, Waltham, MA, USA). Gene expression was analyzed by RT-qPCR using TaqMan™ Gene Expression Assays with FAM-labeled probes (Applied Biosystems, Thermo Fisher Scientific, Waltham, MA, USA). The analyzed targets included MKI67, SOX2, EZH2, TP53, IL-6, CCL2, CXCL8, BAX, and BCL2. A complete list of probes is provided in Table 1. RT-qPCR reactions were performed using TaqMan™ Universal PCR Master Mix II, no UNG (Applied Biosystems, Thermo Fisher Scientific), following the manufacturer’s guidelines. Amplification and data acquisition were carried out on a CFX96 Real-Time PCR System (Bio-Rad Laboratories, Hercules, CA, USA). Relative gene expression was normalized to the housekeeping gene GAPDH and calculated using the 2^−ΔΔCt^ method.

### 4.10. Immunofluorescence

Cells were fixed with 4% paraformaldehyde for 20 min at room temperature, permeabilized with 0.1% Triton X-100 in PBS for 5 min, and blocked in 3% BSA prepared in TBST for 30 min at room temperature. Primary antibodies were diluted in blocking buffer and incubated overnight at 4 °C. The following rabbit monoclonal antibodies from Cell Signaling Technology (Danvers, MA, USA) were used: Vimentin (#5741, 1:200), p16 INK4A (#92803, 1:200), p21 Waf1p21Waf1/Cip1 (#2947, 1:200), HMGB1 (#6893, 1:200), Lamin B1 (#13435, 1:200), α-SMA (#19245, 1:200), COL1A1 (#72026, 1:200), phospho-Histone H2A.X (Ser139) (#9718, 1:400). After primary incubation, cells were washed three times with TBST and incubated for 1 h at room temperature in the dark with fluorophore-conjugated secondary antibodies: goat anti-mouse IgG (H+L) Alexa Fluor™ 488 and goat anti-rabbit IgG (H+L), cross-adsorbed Alexa Fluor™ 568 (Thermo Fisher Scientific, Waltham, MA, USA). Following three additional washes in TBST, nuclei were counterstained with DAPI (0.2 μg/mL; Thermo Fisher Scientific, Waltham, MA, USA). Fluorescence images were acquired using an MW50 fluorescence inverted microscope (OPTA-TECH, Warsaw, Poland).

### 4.11. Protein Isolation and Western Blot Analysis

After 72 h incubation with treatments, A549 cells were harvested, and total protein was extracted using the Minute™ Total Protein Extraction Kit (Invent Biotechnologies, Plymouth, MN, USA). Protein concentrations were determined using the Pierce™ BCA Protein Assay Kit (Thermo Fisher Scientific, Waltham, MA, USA) and normalized across samples. Protein lysates were denatured at 70 °C for 10 min, and equal amounts of protein (10 µg per lane) were separated by SDS-PAGE using the NuPage™/XCell SureLock™ system (Invitrogen, Thermo Fisher Scientific, Waltham, MA, USA). Proteins were subsequently transferred onto PVDF membranes. Membranes were blocked for 1 h at room temperature in 5% BSA or non-fat dry milk prepared in 1× TBST. BSA was used for phosphoproteins and milk for non-phosphorylated proteins. Membranes were then incubated overnight at 4 °C with rabbit recombinant monoclonal antibodies (Cell Signaling Technology, Danvers, MA, USA): EZH2 (#5246), p53 (#2527), β-actin (#4970), p16 INK4A (#92803), p21 Waf1p21Waf1/Cip1 (#2947), HMGB1 (#6893), Lamin B1 (#13435), and phospho-Histone H2A.X (Ser139) (#9718), all used at a dilution of 1:1000. Following incubation, membranes were washed in TBST and incubated with HRP-conjugated secondary antibodies (Cell Signaling Technology) at a dilution of 1:2000 for 1 h at room temperature. Protein bands were detected using SuperSignal™ West Pico chemiluminescent substrate (Thermo Fisher Scientific) and visualized using the ChemiDoc™ Imaging System (Bio-Rad, Hercules, CA, USA). Band intensities were quantified using ImageJ software (National Institutes of Health, Bethesda, MD, USA).

### 4.12. Cell Cycle Analysis

Cell cycle distribution was evaluated by propidium iodide (PI) staining followed by flow cytometry. Briefly, A549 cells were seeded at a density of 1 × 10^5^ cells per well in 12-well plates and treated with the indicated compound concentrations for 72 h. Cells incubated with 1 μM nocodazole for 16 h served as a positive control. Cells were harvested, washed with PBS, and fixed in cold 70% ethanol at −20 °C for 1 h. Fixed cells were subsequently washed with PBS and incubated with RNase A (Canvax Biotech, Valladolid, Spain) at 100 μg/mL for 1 h at 37 °C, followed by staining with 10 μg/mL PI solution (Sigma-Aldrich Corp., St. Louis, MO, USA). After an additional 30 min incubation at 4 °C, samples were analyzed using a flow cytometer, with 2 × 10^5^ cells measured per sample.

### 4.13. β-Galactosidase Staining

Cellular senescence was assessed using the Senescence β-Galactosidase Staining Kit (MedChemExpress, Monmouth Junction, NJ, USA) according to the manufacturer’s instructions. Briefly, cells were washed with PBS and fixed with the provided fixation solution for 10 min at room temperature. After washing, cells were incubated with freshly prepared β-galactosidase staining solution and maintained for 16 h at 37 °C in a CO_2_-free incubator in humidity. Senescent cells exhibiting blue staining were visualized and imaged using an MW50 inverted microscope (OPTA-TECH, Warsaw, Poland). Quantification was performed by manually calculating the percentage of β-galactosidase-positive (blue) cells relative to the total number of cells in six randomly selected fields per well.

### 4.14. DCFH-DA Assay

Intracellular ROS levels were measured using the ROS Assay Kit (MedChemExpress, Monmouth Junction, NJ, USA) based on the fluorescent probe DCFH-DA. Briefly, cells were treated with the investigated compounds or vehicle control for 4 h, followed by incubation with DCFH-DA working solution according to the manufacturer’s instructions. Intracellular esterases deacetylate DCFH-DA to non-fluorescent DCFH, which is subsequently oxidized by ROS to fluorescent DCF. After incubation, cells were washed to remove excess probe, and fluorescence intensity was measured using a microplate fluorescence reader. ROS levels were expressed as relative fluorescence units normalized to control conditions.

### 4.15. Caspase-3 Assay

Caspase-3 activity was assessed using the Caspase-3 Activity Assay Kit (MedChemExpress, Monmouth Junction, NJ, USA) according to the manufacturer’s instructions. A549 cells were seeded at a density of 5 × 10^5^ cells per well in 6-well plates and treated with the indicated compound concentrations for 48 h. Cells incubated with 1 μM staurosporine for 16 h served as a positive control. After incubation, cells were lysed using the provided lysis buffer, and equal volumes of cell lysates were transferred to a 96-well plate. Caspase-3 substrate solution (Ac-DEVD-pNA) was added, and samples were incubated at 37 °C for 2 h to allow enzymatic cleavage of the substrate. Absorbance was measured using a microplate spectrophotometer according to the manufacturer’s protocol. Caspase-3 activity was expressed as a percentage relative to the positive control or normalized to control conditions.

### 4.16. Statistical Analysis

Statistical analyses were performed using GraphPad Prism 11 Academic Pro software. Data are presented as mean ± SEM from at least three independent biological replicates unless otherwise stated. Differences between groups were primarily evaluated using one-way analysis of variance (ANOVA) followed by Tukey’s post hoc test. In cases where the assumption of homogeneity of variance was not met, Brown-Forsythe and Welch corrections were applied. For sphere formation and β-galactosidase assays, non-parametric Kruskal–Wallis tests were applied. Two-way ANOVA was used for the analysis of cell cycle distribution and wound healing assays. Statistical significance was defined as *p* < 0.05.

## 5. Conclusions

The present study demonstrates that pharmacological targeting of EZH2 promotes a cytostatic response associated with features of senescence-associated phenotypic remodeling in lung adenocarcinoma A549 cells, accompanied by activation of cytokine signaling consistent with a senescence-associated secretory phenotype-like response and modulation of tumor–stromal communication without evidence of apoptotic pathway activation. These findings identify EZH2 as a central epigenetic regulator coordinating proliferative capacity, stemness-associated features, and senescence-linked transcriptional programs in NSCLC cells.

Importantly, comparative analysis of catalytic inhibition and targeted proteasomal degradation revealed that EZH2 degradation by MS1943 elicited a more pronounced pro-senescent and anti-proliferative response than enzymatic inhibition alone. This observation supports the concept that non-canonical scaffold functions of EZH2 contribute substantially to tumor cell plasticity and may limit the efficacy of catalytic inhibitors that preserve structural PRC2-associated regulatory activity.

At the microenvironmental level, induction of cytokines associated with senescence-related secretory signaling together with fibroblast activation markers indicates that EZH2-dependent chromatin regulation participates in shaping paracrine signaling networks linking tumor cells with stromal compartments. These data suggest that EZH2-targeting strategies influence not only intrinsic tumor cell behavior but also extracellular regulatory circuits relevant to disease progression and therapeutic response.

Taken together, these findings support the concept that targeted EZH2 degradation represents a mechanistically distinct epigenetic strategy capable of modulating tumor cell plasticity and promoting cytostatic phenotypic remodeling in lung adenocarcinoma cells. In this framework, modulation of EZH2 activity emerges as a promising strategy for controlling tumor cell plasticity and senescence-associated signaling in NSCLC, providing a rationale for further investigation of degrader-based approaches in combination-oriented therapeutic settings.

## Figures and Tables

**Table 1:** TaqMan™ gene expression assays used for RT-qPCR.

Gene Symbol	Gene Name	Assay ID (Applied Biosystems)	Fluorophore
*MKI67*	Marker of proliferation Ki-67	Hs04260396_g1	FAM
*SOX2*	SRY-box transcription factor 2	Hs01053049_s1	FAM
*EZH2*	Enhancer of zeste homolog 2	Hs00544830_m1	FAM
*TP53*	Tumor protein p53	Hs01034249_m1	FAM
*IL-6*	Interleukin 6	Hs00985639_m1	FAM
*CCL2*	C-C motif chemokine ligand 2	Hs00234140_m1	FAM
*CXCL8*	Interleukin 8	Hs00174103_m1	FAM
*BAX*	BCL2-associated X protein	Hs00180269_m1	FAM
*BCL2*	B-cell lymphoma 2	Hs00608023_m1	FAM
*GAPDH*	Glyceraldehyde-3-phosphate dehydrogenase	Hs02786624_g1	FAM

## Data Availability

The data that support the findings of this study are available from the corresponding authors upon reasonable request.
